# Initial sedation strategy and clinical outcomes in mechanically ventilated neurocritical care patients with acute brain injury: a retrospective cohort study

**DOI:** 10.3389/fmed.2026.1833644

**Published:** 2026-08-05

**Authors:** Chunhao Zhou, Daxing Wu, Honghua Guo, Qianliang Huang

**Affiliations:** Department of Neurosurgery, Ganzhou People’s Hospital, Ganzhou Hospital-Nanfang Hospital, Southern Medical University, Ganzhou, Jiangxi, China

**Keywords:** acute brain injury, ICU admission, mechanical ventilation, midazolam, neurocritical care, propofol, sedation

## Abstract

**Background:**

Sedation is a cornerstone in the management of neurocritical care patients with brain injuries. However, optimal sedation practices remain controversial. This study aims to evaluate the association between initial sedation strategies and key clinical outcomes, including duration of mechanical ventilation, ICU length of stay, extubation success, and mortality.

**Methods:**

This retrospective observational study analyzed 147 adult patients with acute brain injury admitted to neurocritical care units. Eligible patients received invasive mechanical ventilation (IMV) for ≥24 h and underwent at least one weaning attempt. Sedation agents assessed included propofol, midazolam, combined regimens, dexmedetomidine, sodium thiopental and no sedation. Data on demographics, comorbidities, ventilation parameters, and outcomes were extracted and analyzed using SPSS v27. Regression models evaluated the impact of sedation type on clinical outcomes.

**Results:**

This study included 147 critically ill patients. Sedation practices shifted over time, with propofol use decreasing from 46.2% on Day 1 to 26.3% on Day 7, while the proportion of patients receiving no sedation increased to 38.7%. Hospital interventions, including use of ICP monitors and ventilation strategies, varied significantly across sedation groups. Combined sedation and no sedation groups had significantly prolonged mechanical ventilation and ICU stays. Regression analysis showed that compared to propofol, combined sedation was associated with increased IMV days (B = 3.470, *p* < 0.001), ICU stay (B = 5.501, *p* < 0.001), and delayed extubation (B = 5.662, *p* < 0.001). Midazolam and no-sedation groups showed variable associations across outcomes. These findings highlight the impact of sedation strategy on patient recovery and ICU resource utilization.

**Conclusion:**

Sedation strategy significantly influences clinical aspects in neurocritical care. Propofol appears superior for facilitating faster recovery, while midazolam and combined use are linked to adverse outcomes. Individualized, protocol-driven sedation approaches are essential to optimize care for patients with acute brain injury.

## 1 Introduction

Acute brain injury has an increasingly high prevalence with subsequent burdens on individuals, families and health care systems (1). It often necessitates neurocritical admission with a substantial need for invasive ventilation and sedation to ensure patient comfort, facilitate ventilator synchrony, and control intracranial pressure (ICP) (2, 3).

Sedatives and analgesics such as propofol, midazolam, dexmedetomidine are routinely used to manage pain, anxiety, agitation, and elevated ICP in this population (4, 5). However, excess or prolonged sedation is known to have adverse effects: deep early sedation prolongs ventilation and ICU stay, and is associated with higher delirium rates and mortality (2, 6, 7).

The Society of Critical Care Medicine (SCCM) guidelines highlight that lighter sedation targets lead to shorter extubation times, fewer ICU days, and reduced complications (3, 8). Additionally, an international prospective cohort study reported that elevated sedation levels were linked to both a delayed start of the weaning process and an increased risk of weaning failure (6, 9). Despite the current expanding research, sedation management is still challenging, especially with mechanically ventilated patients. Expert consensus notes that insufficient evidence and guidelines specific to neurologic injury contribute to wide variation in practice. Neurocritical care experts advocate an “analgosedation” approach (prioritizing analgesia before sedatives) and emphasize tailoring agent selection to each patient (10, 11). A systematic review comparing propofol and midazolam in adult ICU patients found that propofol use was linked to shorter ICU stays, reduced duration of mechanical ventilation, and faster extubation in patients undergoing acute surgical care (12). However, recent cohort study involving approximately 1,450 patients with brain injury requiring mechanical ventilation and a review by Ostermann et al., showed that there was no significant difference regarding mechanical ventilation parameters between these sedatives in critically ill patients (13, 14).

The impact of initial sedation choice on clinical outcomes in neurocritical patients is unclear. While sedation is necessary for many patients, it may delay neurological assessments and recovery. Conversely, inadequate sedation risks agitation or ICP surges. Despite these insights, the evidence about sedation protocols is still controversial. This study aims to evaluate how initial sedation strategies influence duration of mechanical ventilation, ICU length of stay, neurologic recovery, and mortality in adult patients with acute brain injury.

## 2 Materials and methods

### 2.1 Study design and setting

This retrospective observational cohort study analyzed adult patients admitted to the neurocritical care units. Data were obtained from the institutional electronic health record system. The study was conducted in accordance with the principles of the Declaration of Helsinki and was approved by the Institutional Review Board of Ganzhou People’s Hospital. All patient identifiers were removed before analysis to ensure confidentiality.

### 2.2 Study population

Adult patients admitted with acute brain injury requiring invasive mechanical ventilation were screened for eligibility. Patients were included if they were aged 18 years or older, required invasive mechanical ventilation for at least 24 h, had a documented diagnosis of acute brain injury such as traumatic brain injury, aneurysmal subarachnoid hemorrhage, intracerebral hemorrhage, or ischemic stroke, and had a pre-intubation Glasgow Coma Scale (GCS) score of 12 or less. In addition, patients were required to have undergone at least one attempt at ventilator weaning during their ICU stay. Patients were excluded if they had pre-existing neuromuscular disorders affecting respiratory function, including myasthenia gravis or Guillain-Barré syndrome, major traumatic chest injuries interfering with respiratory mechanics, pre-existing tracheostomy prior to ICU admission, or high cervical spinal cord injury affecting respiratory drive. Patients with incomplete clinical data relevant to sedation or outcome variables were also excluded.

### 2.3 Sedation exposure

Sedation strategy was determined according to the primary sedative agent administered during the first 24 h of ICU admission (Day 1). Sedative agents evaluated in this study included propofol, midazolam, dexmedetomidine, sodium thiopental, combined sedation regimens, and no sedation. Combined sedation referred to the concurrent administration of more than one sedative agent. Sedation practices were also assessed longitudinally on Days 3 and 7 of ICU admission in order to describe trends in sedation management over time.

### 2.4 Data collection

Demographic and clinical data were retrospectively extracted from the electronic medical records. Baseline variables included patient age, sex, body mass index (BMI), smoking status, and underlying cause of brain injury. Comorbid conditions such as diabetes mellitus, hypertension, chronic obstructive pulmonary disease, heart failure, malignancy, and central nervous system infections were also recorded. Clinical and physiological parameters documented on the first day of sedation included the Glasgow Coma Scale score and motor component (GCS-M), Richmond Agitation-Sedation Scale (RASS) score, respiratory rate, PaO2/FiO2 ratio, plateau pressure, intracranial pressure values among monitored patients, and the use of intracranial pressure monitoring. Analgesic use was also recorded. Additional hospital-related variables such as the need for neurosurgical interventions, including decompressive craniotomy, and the type of mechanical ventilation mode were also collected.

### 2.5 Outcome measures

The primary outcomes of the study were the duration of invasive mechanical ventilation and the length of stay in the intensive care unit. Secondary outcomes included time to first extubation, extubation failure, extubation failure within 72 h, RASS score on the day of extubation, ICU mortality, and overall in-hospital mortality. Additional clinical outcomes and complications were documented, including the occurrence of ventilator-associated pneumonia, acute respiratory distress syndrome, the need for tracheostomy, and episodes of anisocoria during hospitalization. Ventilator parameters recorded at the time of extubation included positive end-expiratory pressure, oxygen saturation, and tidal volume.

### 2.6 Statistical analysis

Statistical analysis was performed using SPSS software (version 27.0, IBM Corp., Armonk, NY). Continuous variables were summarized as medians with interquartile ranges due to non-normal distribution, while categorical variables were expressed as frequencies and percentages. The Shapiro–Wilk test was used to assess the normality of data distribution. Differences between sedation groups were evaluated using the Kruskal-Wallis test for continuous variables and the chi-square test for categorical variables. Multivariable linear regression models were constructed to assess the association between initial sedation strategy and clinical outcomes, using propofol as the reference category. Regression analyses were restricted to patients receiving propofol, midazolam, or combined sedation during the first day of ICU admission. Patients receiving dexmedetomidine or sodium thiopental were excluded from regression analysis due to limited sample size and incomplete outcome data. Missing data were handled using pairwise deletion, and no imputation methods were applied. A two-sided *p*-value of less than 0.05 was considered statistically significant (15).

## 3 Results

### 3.1 Demographic characteristics

The demographic and baseline neurologic characteristics (Table 1) of patients stratified by sedation method on Day 1 showed no statistically significant differences across groups regarding age, BMI, gender, smoking status, COPD, heart failure, diabetes mellitus, malignancy, aneurysmal subarachnoid hemorrhage, and intracranial hemorrhage. However, statistically significant differences were noted for initial GCS (*p* = 0.018), initial GCS-M (*p* = 0.021), Day 1 RASS score (*p* < 0.001), hypertension (*p* = 0.036), traumatic brain injury (*p* = 0.003), and ischemic stroke (*p* < 0.001), indicating clinical variation across Day 1 sedation groups.

**TABLE 1:** Demographic characteristics of included patients stratified by day 1 sedation.

Variable	Propofol *n* = 67	Midazolam *n* = 36	Propofol + midazolam *n* = 24	Dexmedetomidine *n* = 2	Sodium thiopental *n* = 1	None *n* = 15	*P*-value
Age, years, median (Q1, Q3)	51 (35, 63)	52.5 (45, 61.5)	53 (42, 64.5)	48.5 (46, 51)	67 (67, 67)	55 (33, 70)	0.635
BMI, kg/m^2^, median (Q1, Q3)	27 (23.45, 29.4)	26.9 (23.4, 28.4)	28.4 (26, 31.4)	26.1 (26.1, 26.1)	21.2 (21.2, 21.2)	27.5 (22.5, 29.8)	0.391
Initial GCS, median (Q1, Q3)	7 (5, 9)	6 (4, 8)	5 (3, 7)	8 (7, 9)	4 (4, 4)	9 (7, 11)	0.018
Initial GCS-M, median (Q1, Q3)	4 (3, 5)	3 (2, 5)	3 (2, 4)	5 (4, 5)	2 (2, 2)	5 (4, 6)	0.021
Day 1 RASS score, median (Q1, Q3)	−3 (−4, −2)	−4 (−5, −3)	−4 (−5, −4)	−2 (−2, −1)	−5 (−5, −5)	0 (−1, 0)	<0.001^a^
Pre-intubation GCS ≤ 12, *n*/*N* (%)	67/67 (100)	36/36 (100)	24/24 (100)	2/2 (100)	1/1 (100)	15/15 (100)	–
Female, *n*/*N* (%)	22/67 (32.8)	11/36 (30.6)	9/24 (37.5)	2/2 (100)	0/1 (0)	8/15 (53.3)	0.436
Male, *n*/*N* (%)	45/67 (67.2)	25/36 (69.4)	15/24 (62.5)	0/2 (0)	1/1 (100)	7/15 (46.7)	–
Smoking, yes, *n*/*N* (%)	18/67 (26.9)	10/36 (27.8)	6/24 (25.0)	1/2 (50.0)	0/1 (0)	3/15 (20.0)	0.830
COPD, yes, *n*/*N* (%)	4/67 (6.0)	2/36 (5.6)	2/24 (8.3)	0/2 (0)	0/1 (0)	0/15 (0)	0.705
Heart failure, yes, *n*/*N* (%)	3/67 (4.5)	3/36 (8.3)	1/24 (4.2)	0/2 (0)	0/1 (0)	0/15 (0)	0.627
Hypertension, yes, *n*/*N* (%)	24/67 (35.8)	7/36 (19.4)	5/24 (20.8)	0/2 (0)	1/1 (100)	8/15 (53.3)	0.036
Diabetes mellitus, yes, *n*/*N* (%)	9/67 (13.4)	8/36 (22.2)	4/24 (16.7)	0/2 (0)	0/1 (0)	3/15 (20.0)	0.784
Malignancy, yes, *n*/*N* (%)	3/67 (4.5)	2/36 (5.6)	2/24 (8.3)	0/2 (0)	0/1 (0)	2/15 (13.3)	0.656
Traumatic brain injury, yes, *n*/*N* (%)	24/67 (35.8)	26/36 (72.2)	17/24 (70.8)	2/2 (100)	1/1 (100)	8/15 (53.3)	0.003^a^
Aneurysmal subarachnoid hemorrhage, yes, *n*/*N* (%)	11/67 (16.4)	5/36 (13.9)	5/24 (20.8)	1/2 (50.0)	0/1 (0)	1/15 (6.7)	0.583
Intracranial hemorrhage, yes, *n*/*N* (%)	26/67 (38.8)	6/36 (16.7)	10/24 (41.7)	0/2 (0)	0/1 (0)	6/15 (40.0)	0.066
Ischemic stroke, yes, *n*/*N* (%)	5/67 (7.5)	1/36 (2.8)	1/24 (4.2)	0/2 (0)	0/1 (0)	6/15 (40.0)	<0.001^a^
CNS infection, yes, *n*/*N* (%)	2/67 (3.0)	2/36 (5.6)	1/24 (4.2)	0/2 (0)	1/1 (100)	2/15 (13.3)	0.422
Brain tumor, yes, *n*/*N* (%)	3/67 (4.5)	2/36 (5.6)	2/24 (8.3)	0/2 (0)	0/1 (0)	2/15 (13.3)	0.688

Continuous variables are presented as median (Q1, Q3). Categorical variables are presented as *n*/*N* (%). BMI, body mass index; COPD, chronic obstructive pulmonary disease; CNS, central nervous system; GCS, Glasgow Coma Scale; GCS-M, Glasgow Coma Scale motor component; RASS, Richmond Agitation-Sedation Scale.

^a^Statistically significant < 0.05.

### 3.2 Sedation practices

**FIGURE 1:**
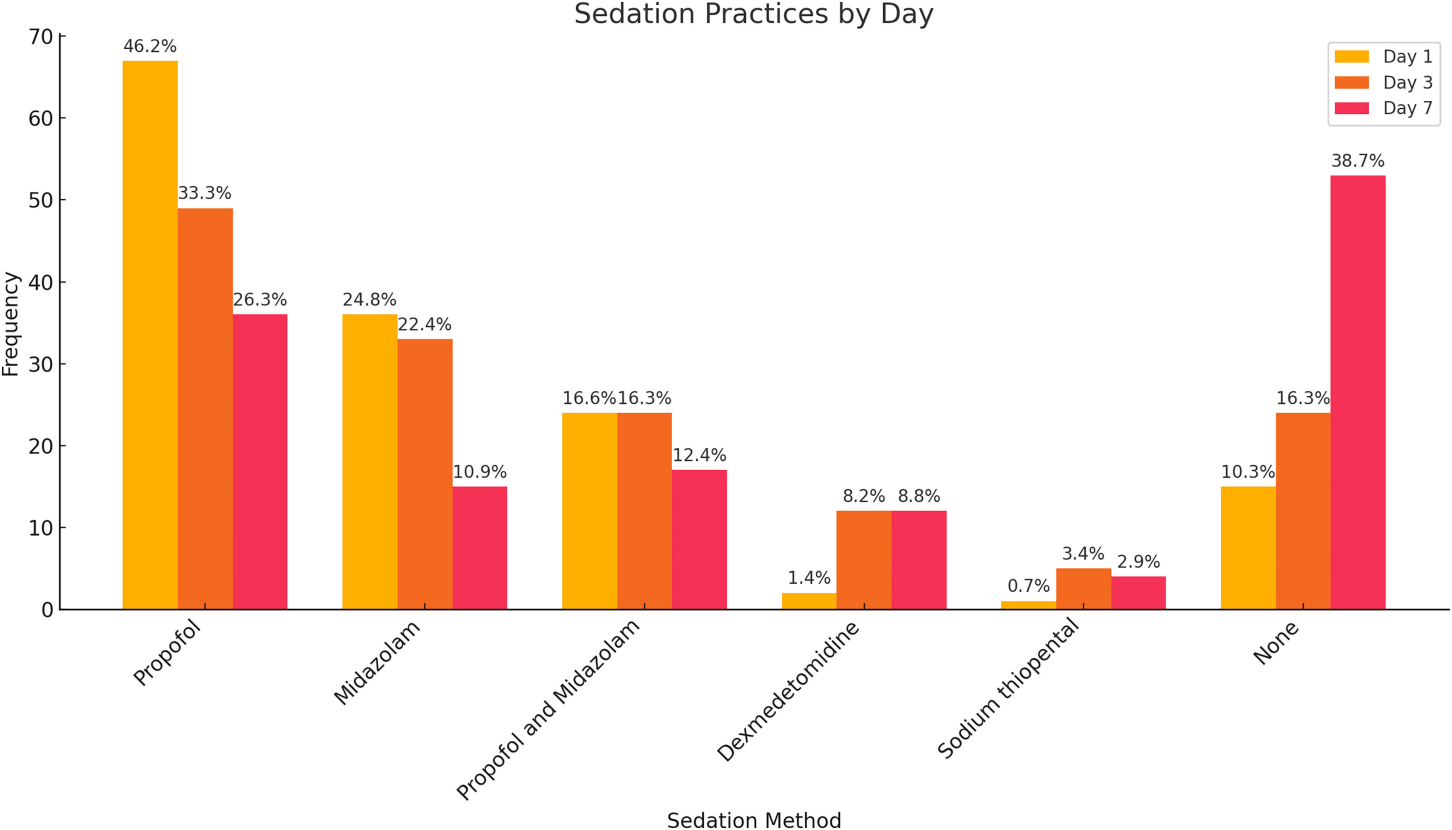
Grouped bar chart illustrating the sedation practices across Day 1, Day 3, and Day 7.

Sedation practices varied significantly among patients from Day 1 to Day 7. As illustrated in Figure 1, propofol was the most frequently used sedative on Day 1, administered to 46.2% of patients, but its usage steadily decreased to 33.3% on Day 3 and further declined to 26.3% by Day 7. In contrast, the proportion of patients receiving no sedation increased markedly from 10.3% on Day 1 to 38.7% by Day 7.

### 3.3 Hospital characteristics

Hospital characteristics (Table 2) also demonstrated significant differences across sedation methods. Initial ICP among monitored patients (*p* = 0.042), maximum ICP within the first 24 h (*p* = 0.033), opioid analgesic use (*p* = 0.012), Day 1 RASS score (*p* < 0.001), and respiratory rate on Day 1 (*p* < 0.001) differed significantly among groups. The use of ICP monitors (*p* = 0.001), decompressive craniotomy (*p* = 0.031), Day 1 ventilation (*p* = 0.020), pressure-assist ventilation (*p* = 0.014), and volume-assist ventilation (*p* = 0.003) were also significantly associated with sedation practice, indicating variation in management strategies across sedation groups.

**TABLE 2:** Hospital characteristics of included patients stratified by day 1 sedation.

Variable	Propofol *n* = 67	Midazolam *n* = 36	Propofol + midazolam *n* = 24	Dexmedetomidine *n* = 2	Sodium thiopental *n* = 1	None *n* = 15	*P*-value
Day 1 PaO2, median (Q1, Q3)	117 (108.5, 124.5)	119 (114, 122)	119 (112, 124)	128 (121, 135)	142 (142, 142)	113 (104, 128)	0.739
Day 1 FiO2, median (Q1, Q3)	41.7 (38.4, 44.2)	42.2 (36.6, 45.8)	44.3 (38.2, 45.7)	41.4 (41, 41.7)	65.5 (65.5, 65.5)	37.9 (37.15, 39.45)	0.092
Day 1 plateau pressure, cmH2O, median (Q1, Q3)	19 (17, 22)	20 (18, 23)	21 (19, 24)	18 (17, 19)	24 (24, 24)	18 (16, 21)	0.071
Initial ICP among monitored patients, mmHg, median (Q1, Q3)	18 (14, 23)	21 (17, 26)	23 (18, 29)	16 (15, 17)	28 (28, 28)	17 (13, 22)	0.042
Maximum ICP within first 24 h, mmHg, median (Q1, Q3)	24 (19, 31)	28 (22, 35)	31 (25, 39)	22 (20, 24)	36 (36, 36)	23 (18, 30)	0.033
Opioid analgesic use, *n*/*N* (%)	49/67 (73.1)	29/36 (80.6)	21/24 (87.5)	1/2 (50.0)	1/1 (100)	6/15 (40.0)	0.012
Non-opioid analgesic use, *n*/*N* (%)	25/67 (37.3)	13/36 (36.1)	10/24 (41.7)	1/2 (50.0)	0/1 (0)	7/15 (46.7)	0.781
Day 1 RASS score, median (Q1, Q3)	−3 (−4, −2)	−4 (−5, −3)	−4 (−5, −4)	−2 (−2, −1)	−5 (−5, −5)	0 (−1, 0)	<0.001^a^
Day 1 respiratory rate, median (Q1, Q3)	16 (15, 17)	17 (16, 18)	17 (17, 18)	18 (18, 18)	16 (16, 16)	19 (17, 20)	<0.001^a^
ICP monitor, yes, *n*/*N* (%)	23/67 (34.3)	23/36 (63.9)	17/24 (70.8)	1/2 (50.0)	1/1 (100)	4/15 (26.7)	0.001^a^
External ventricular drain, yes, *n*/*N* (%)	26/67 (38.8)	9/36 (25.0)	7/24 (29.2)	1/2 (50.0)	0/1 (0)	5/15 (33.3)	0.421
Neurosurgery, yes, *n*/*N* (%)	33/67 (49.3)	11/36 (30.6)	8/24 (33.3)	2/2 (100)	0/1 (0)	8/15 (53.3)	0.204
Decompressive craniotomy, yes, *n*/*N* (%)	5/67 (7.5)	10/36 (27.8)	6/24 (25.0)	1/2 (50.0)	0/1 (0)	2/15 (13.3)	0.031
Barbiturate coma, yes, *n*/*N* (%)	2/67 (3.0)	0/36 (0)	2/24 (8.3)	0/2 (0)	1/1 (100)	0/15 (0)	0.245
Day 1 ventilation, yes, *n*/*N* (%)	6/67 (9.0)	5/36 (13.9)	3/24 (12.5)	0/2 (0)	0/1 (0)	6/15 (40.0)	0.020
Pressure assist, yes, *n*/*N* (%)	5/67 (7.5)	5/36 (13.9)	3/24 (12.5)	0/2 (0)	0/1 (0)	6/15 (40.0)	0.014
Pressure control, yes, *n*/*N* (%)	10/67 (14.9)	2/36 (5.6)	4/24 (16.7)	0/2 (0)	0/1 (0)	2/15 (13.3)	0.537
Spontaneous breathing, yes, *n*/*N* (%)	1/67 (1.5)	1/36 (2.8)	2/24 (8.3)	0/2 (0)	0/1 (0)	1/15 (6.7)	0.400
Volume assist, yes, *n*/*N* (%)	48/67 (71.6)	33/36 (91.7)	19/24 (79.2)	1/2 (50.0)	1/1 (100)	6/15 (40.0)	0.003^a^

Continuous variables are presented as median (Q1, Q3). Categorical variables are presented as *n*/*N* (%). ICP, intracranial pressure; PaO2, arterial oxygen partial pressure; FiO2, inspired oxygen fraction; RASS, Richmond Agitation-Sedation Scale.

^a^Statistically significant < 0.05.

### 3.4 Outcomes

Patient outcomes (Table 3) showed significant differences in RASS score on the day of extubation (*p* = 0.044), duration of invasive mechanical ventilation (IMV) (*p* < 0.001), ICU length of stay (*p* < 0.001), and days to first extubation (*p* < 0.001). ICU mortality (*p* = 0.029) and hospital mortality (*p* = 0.004) were significantly associated with sedation practices, with the highest hospital mortality rate in the non-sedated group. Extubation failure and extubation failure within 72 h were not significantly different across groups (both *p* = 0.766). Nosocomial ventilator-associated pneumonia (VAP) also significantly differed among groups (*p* = 0.001), highlighting higher infection rates in sedated patients compared to those without sedation.

**TABLE 3:** Outcomes of included patients stratified by day 1 sedation.

Variable	Propofol *n* = 67	Midazolam *n* = 36	Propofol + midazolam *n* = 24	Dexmedetomidine *n* = 2	Sodium thiopental *n* = 1	None *n* = 15	*P*-value
Extubation tidal volume, median (Q1, Q3)	486 (466, 512)	493 (477, 520)	490 (461.5, 513)	N/A	481 (481, 481)	490 (476, 516)	0.634
Extubation PEEP, median (Q1, Q3)	5.53 (5.32, 5.8)	5.47 (5.22, 5.65)	5.54 (5.31, 5.81)	N/A	N/A	5.49 (5.28, 5.61)	0.548
Extubation SpO2, median (Q1, Q3)	98 (97, 99)	98 (98, 99)	98 (97, 98)	98 (97, 99)	97 (97, 97)	99 (98, 99)	0.077
RASS score on day of extubation, median (Q1, Q3)	0 (−1, 0)	−1 (−2, 0)	−1 (−2, 0)	0 (0, 0)	−2 (−2, −2)	0 (0, 0)	0.044
IMV days, median (Q1, Q3)	7 (5, 7)	6 (5, 8)	10 (8, 12)	5 (5, 5)	18 (18, 18)	6.5 (6, 7)	<0.001^a^
ICU length of stay, days	12 (12, 15)	16 (15, 18)	18 (16, 20)	7.5 (7, 8)	48 (48, 48)	14 (13, 18)	<0.001^a^
Days to first extubation	5 (4, 6)	7 (6, 8)	11 (9, 14)	1 (1, 1)	10 (10, 10)	5 (4, 6)	<0.001^a^
ICU mortality, yes, *n*/*N* (%)	6/67 (9.0)	10/36 (27.8)	4/24 (16.7)	0/2 (0)	1/1 (100)	0/15 (0)	0.029
Hospital mortality, yes, *n*/*N* (%)	4/67 (6.0)	1/36 (2.8)	0/24 (0)	0/2 (0)	1/1 (100)	4/15 (26.7)	0.004^a^
Extubation failure, yes, *n*/*N* (%)	17/67 (25.4)	9/36 (25.0)	4/24 (16.7)	1/2 (50.0)	1/1 (100)	4/15 (26.7)	0.766
Extubation failure within 72 h, yes, *n*/*N* (%)	17/67 (25.4)	9/36 (25.0)	4/24 (16.7)	1/2 (50.0)	1/1 (100)	4/15 (26.7)	0.766
Tracheostomy, yes, *n*/*N* (%)	22/67 (32.8)	8/36 (22.2)	8/24 (33.3)	2/2 (100)	1/1 (100)	8/15 (53.3)	0.236
ARDS, yes, *n*/*N* (%)	1/67 (1.5)	1/36 (2.8)	2/24 (8.3)	0/2 (0)	0/1 (0)	1/15 (6.7)	0.392
Nosocomial VAP, yes, *n*/*N* (%)	16/67 (23.9)	19/36 (52.8)	16/24 (66.7)	1/2 (50.0)	1/1 (100)	7/15 (46.7)	0.001^a^
Episodic anisocoria, yes, *n*/*N* (%)	17/67 (25.4)	7/36 (19.4)	12/24 (50.0)	0/2 (0)	0/1 (0)	6/15 (40.0)	0.059

Continuous variables are presented as median (Q1, Q3). Categorical variables are presented as *n*/*N* (%). ARDS, acute respiratory distress syndrome; IMV, invasive mechanical ventilation; ICU, intensive care unit; PEEP, positive end-expiratory pressure; RASS, Richmond Agitation-Sedation Scale; SpO2, peripheral oxygen saturation; VAP, ventilator-associated pneumonia.

^a^Statistically significant < 0.05.

### 3.5 Regression analysis

Regression analysis using propofol as the reference group revealed significant predictors for increased days on invasive mechanical ventilation (IMV) and ICU length of stay. For IMV days, midazolam (B = 0.206, *p* = 0.708) showed no significant difference compared to propofol. However, combined sedation methods (B = 3.470, *p* < 0.001) and no sedation (B = 1.830, *p* = 0.017) significantly increased IMV duration. Regarding ICU length of stay, midazolam (B = 3.556, *p* < 0.001), combined sedation methods (B = 5.501, *p* < 0.001), and no sedation (B = 2.330, *p* = 0.034) were all significantly associated with prolonged stays compared to propofol. Finally, days to first extubation were significantly longer in patients sedated with midazolam (B = 1.531, *p* = 0.004) and combined sedation methods (B = 5.662, *p* < 0.001), whereas no sedation (B = −0.025, *p* = 0.973) showed no significant difference compared to propofol. These findings underscore the importance of sedation choice on critical patient outcomes (Table 4).

**TABLE 4:** Multiple regression analysis table.

Reference group	Predictor	B coefficient	*P*-value
Reference (Propofol)	Invasive mechanical ventilation days		
	Midazolam	0.206	0.708
	Combined	3.47	<0.001
	None	1.83	0.017
Reference (Propofol)	ICU length of stay		
	Midazolam	3.556	<0.001
	Combined	5.501	<0.001
	None	2.33	0.034
Reference (Propofol)	Time to first extubation		
	Midazolam	1.531	0.004
	Combined	5.662	<0.001
	None	–0.025	0.973

## 4 Discussion

The selection of an appropriate sedative for neurocritically patients should take into account both the potential benefits and risks, along with the specific clinical circumstances. Propofol is widely utilized in ICUs for managing patients with acute brain injury, particularly for controlling ICP. Its sedative effect is dose-dependent: at lower doses (<4 mg/kg/h), it maintains cerebrovascular autoregulation, cerebral blood flow (CBF)/metabolic rate (CMRO2) coupling, and adequate brain oxygenation. At higher doses (>5 mg/kg/h), it can induce EEG burst suppression, which is beneficial in the treatment of neurological insults (16). On the other hand, midazolam, which has a relatively short half-life of approximately 1 h, is highly lipophilic predisposing it to tissue accumulation during prolonged infusion. This can result in delayed awakening and hinder accurate neurological assessment. The extent of delayed recovery varies significantly between individuals (17).

The combination of propofol and midazolam is commonly used as a first-line sedative regimen in patients with acute brain injury. However, their use varies across clinicians and countries, influenced by individual practice preferences and cost considerations (18). Dexmedetomidine provides sedative effects with preserved arousability, and is associated with faster extubation, fewer delirium and coma days, lower delirium rates, and reduced mortality in some ICU patients. However, hypotension and bradycardia are common side effects (19). Despite these findings, the literature on sedation is heterogeneous and contains conflicting evidence. Hence, optimizing sedation in patients with acute brain injury is crucial.

The present study highlights crucial insights into sedation practices and their associations with clinical outcomes for critically ill patients in neurocritical care. Among 147 patients with acute brain injury involved in this study, demographic characteristics such as age, BMI, and comorbidities like COPD and diabetes mellitus were similar across sedation groups, with observed significant differences in baseline neurologic scores, hypertension, traumatic brain injury, and ischemic stroke.

The descriptive analysis of sedation trends observed in our study revealed a substantial decline in the use of propofol over time, with a reciprocal increase in patients receiving no sedation by the 7^th^ day. This transition aligns with current clinical guidelines advocating for light or no sedation to facilitate early awakening, reduce delirium risk, and improve ventilator weaning (3).

In spite of the increased use of minimal sedation, it appeared paradoxically associated with higher ICU and hospital mortality. This counterintuitive result could be explained by the possibility that patients deemed too unstable or non-responsive may not have received sedation due to already poor prognosis (20). Hospital-level interventions also varied with sedation status, with non-sedated patients having higher respiratory rates and lower utilization of invasive monitoring and supportive procedures. This pattern suggests a different approach to care and possibly severity of illness, reinforcing the need for standardized sedation protocols to minimize inter-institutional variability (21).

Based on our findings, sedation type was significantly associated with key clinical endpoints. Patients receiving combined sedatives or midazolam experienced longer durations on invasive mechanical ventilation (IMV), extended ICU stays, and delayed extubation. These results corroborate existing literature, which associates benzodiazepine-based sedation, particularly midazolam, with prolonged ICU stay and higher risks of delirium and VAP (22). On the other hand, our findings contrast with the recent study by Feng et al., which found no difference between sedatives in the duration of mechanical ventilation, but are consistent with its finding of a longer ICU stay associated with midazolam (13).

Interestingly, the no sedation group did not significantly differ from the propofol group in extubation timing and incidence of extubation failure, implying that, under appropriate clinical conditions, withholding sedation might support faster recovery without compromising airway management. Regression analyses further underscore the deleterious impact of combined sedation and midazolam use, with significantly increased durations of IMV and ICU stay. Propofol alone was consistently associated with more favorable outcomes, consistent with its pharmacokinetic profile that allows for rapid titration and short recovery times (23). These findings support the preferential use of propofol in neurocritical care when sedation is necessary and reinforce the shift toward daily sedation interruption or light sedation strategies (24). Moreover, the higher rates of VAP among sedated patients may reflect impaired airway clearance and reduced mobility due to deep sedation, further arguing for conservative sedation use (25). Although episodic anisocoria was more frequent among patients who did not receive sedation, the difference was not statistically significant.

Some limitations should be acknowledged, and caution should be exercised when interpreting the results of this study. First, a methodological limitation of this study was its retrospective design. Additionally, the sample size within each treatment group was relatively small, which may limit the generalizability of the results. The limited number of patients who received sodium thiopental and/or dexmedetomidine prevented us from drawing meaningful conclusions about these groups. Additionally, our findings are restricted to the impact of initial sedation strategies on patient outcomes and do not account for any subsequent changes in sedative type. Finally, the use of multivariate analysis should be approached with caution due to the limited number of participants.

## 5 Conclusion

In conclusion, this study underscores the pivotal role of initial sedation strategies in managing critically ill patients with acute brain injury. Our findings demonstrate that propofol, when used alone, was consistently associated with more favorable outcomes, including shorter durations of mechanical ventilation, reduced ICU stay, and timely extubation. In contrast, midazolam and combined sedative regimens were linked to prolonged recovery and increased risks of complications such as ventilator-associated pneumonia. Patients who received no sedation showed higher mortality rates. These results confirm the importance of individualized sedation plans and suggest that lighter sedation or daily interruptions, guided by clinical status, may enhance recovery while minimizing adverse effects. Therefore, standardized protocols tailored to neurocritical populations are urgently needed. Further prospective, multicenter trials should aim to refine these protocols and optimize sedation practices for improved patient outcomes.

## Data Availability

The raw data supporting the conclusions of this article will be made available by the authors, without undue reservation.
